# High rates of hospital admission among older residents in assisted living facilities: opportunities for intervention and impact on acute care

**Published:** 2014-03-04

**Authors:** David B Hogan, Joseph E Amuah, Laurel A Strain, Walter P Wodchis, Andrea Soo, Misha Eliasziw, Andrea Gruneir, Brad Hagen, Gary Teare, Colleen J Maxwell

**Affiliations:** David B. Hogan, MD, is a Professor and Brenda Strafford Foundation Chair in Geriatric Medicine, University of Calgary, Calgary, Alberta.; Joseph E. Amuah, PhD, is a Senior Researcher with the Health System Performance Branch, Canadian Institute for Health Information, Ottawa, Ontario.; Laurel A. Strain, PhD, is a Professor with the Department of Sociology, University of Alberta, Edmonton, Alberta.; Walter P. Wodchis, PhD, is an Associate Professor with the Institute of Health Policy, Management and Evaluation, University of Toronto; a Research Scientist with the Toronto Rehabilitation Institute; and an Adjunct Scientist with the Institute for Clinical Evaluative Sciences, Toronto, Ontario.; Andrea Soo, MSc, is a PhD candidate with the Department of Community Health Sciences, University of Calgary, Calgary, Alberta.; Misha Eliasziw, PhD, is an Associate Professor with the Department of Public Health and Community Medicine, Tufts University, Boston, Massachusetts.; Andrea Gruneir, PhD, is a Scientist with the Women's College Research Institute, Women's College Hospital, University of Toronto; an Assistant Professor in the Institute of Health Policy, Management and Evaluation, University of Toronto; and an Adjunct Scientist with the Institute for Clinical Evaluative Sciences, Toronto, Ontario.; Brad Hagen, PhD, is an Associate Professor with the Faculty of Health Sciences, University of Lethbridge, Lethbridge, Alberta.; Gary Teare, PhD, is Director of Measurement and Analysis, Saskatchewan Health Quality Council, and an Adjunct Professor with the College of Medicine and School of Public Health, University of Saskatchewan, Saskatoon, Saskatchewan.; Colleen J. Maxwell, PhD, is a Professor with the Schools of Pharmacy and of Public Health and Health Systems, University of Waterloo, Waterloo, Ontario, and an Adjunct Scientist with the Institute for Clinical Evaluative Sciences, Toronto, Ontario.

## Abstract

**Background:**

Little is known about health or service use outcomes for residents of Canadian assisted living facilities. Our objectives were to estimate the incidence of admission to hospital over 1 year for residents of designated (i.e., publicly funded) assisted living (DAL) facilities in Alberta, to compare this rate with the rate among residents of long-term care facilities, and to identify individual and facility predictors of hospital admission for DAL residents.

**Methods:**

Participants were 1066 DAL residents (mean age ± standard deviation 84.9 ± 7.3 years) and 976 longterm care residents (85.4 ± 7.6 years) from the Alberta Continuing Care Epidemiological Studies (ACCES). Research nurses completed a standardized comprehensive assessment for each resident and interviewed family caregivers at baseline (2006 to 2008) and 1 year later. We used standardized interviews with administrators to generate facility- level data. We determined hospital admissions through linkage with the Alberta Inpatient Discharge Abstract Database. We used multivariable Cox proportional hazards models to identify predictors of hospital admission.

**Results:**

The cumulative annual incidence of hospital admission was 38.9% (95% confidence interval [CI] 35.9%– 41.9%) for DAL residents and 13.7% (95% CI 11.5%–15.8%) for long-term care residents. The risk of hospital admission was significantly greater for DAL residents with greater health instability, fatigue, medication use (11 or more medications), and 2 or more hospital admissions in the preceding year. The risk of hospital admission was also significantly higher for residents from DAL facilities with a smaller number of spaces, no licensed practical and/ or registered nurses on site (or on site less than 24 hours a day, 7 days a week), no chain affiliation, and from select health regions.

**Interpretation:**

The incidence of hospital admission was about 3 times higher among DAL residents than among long-term care residents, and the risk of hospital admission was associated with a number of potentially modifiable factors. These findings raise questions about the complement of services and staffing required within assisted living facilities and the potential impact on acute care of the shift from long-term care to assisted living for the facility-based care of vulnerable older people.

Assisted living is a residential option increasingly used by older adults requiring supportive care.^1^,^2^ Assisted living facilities aim to provide secure housing, personal support, and limited health care while promoting autonomy and privacy.^3^ In response to the escalating costs of long-term care facilities (i.e., nursing homes) and older people's preferences for homelike settings, several Canadian provinces have rapidly expanded publicly funded assisted living over recent years.^1^,^4^

In jurisdictions such as Alberta, assisted living is considered an alternative to long-term care for many older adults requiring supportive care.^4^ However, assisted living differs from traditional nursing homes in a number of important ways. Assisted living residents have a high prevalence of chronic illness, disability, and frailty.^5^–^7^ Yet, relative to nursing homes, assisted living facilities are characterized by lower levels of staffing and professional service, which raises questions about their ability to care for more vulnerable older people.^8^–^11^ Delayed detection of emerging health issues and diminished ability to provide augmented care could lead to poorer outcomes for assisted living residents and, ultimately, higher use of acute care.^12^,^13^ When asked to compare assisted living with long-term care, US physicians reported less confidence in the skills of assisted living staff, described fewer treatment options in this setting, and indicated that they were more likely to transfer an assisted living resident with a medical problem to an emergency department.^14^

Current understanding of the place of assisted living in the continuum of supportive housing options for older Canadians is largely extrapolated from US studies.^2^,^5^–^14^ However, the differing structure and function of the Canadian health care systems make this approach problematic. An important outcome for assisted living facilities is the proportion of residents requiring an overnight stay in an acute care setting. Although many of these admissions are necessary, some are potentially avoidable with appropriate and timely care and clinical oversight. Our study objectives were to estimate the incidence of admission to hospital among residents of designated assisted living (DAL) facilities (as described below) in Alberta over the year after each person's baseline assessment, to compare this rate with the rate observed among long-term care residents from the same catchment areas and follow-up period, and to identify characteristics of DAL residents and facilities associated with an increased risk for admission to hospital.

## Methods

### Study design

Data were derived from the Alberta Continuing Care Epidemiological Studies (ACCES), a longitudinal investigation of assisted living and long-term care residents in the province of Alberta, Canada.^15^,^16^ The assisted living cohort consisted of older residents of DAL facilities—i.e., publicly funded assisted living and supportive housing facilities—in 5 former health regions (2 major urban regions and 3 largely rural regions). At the time of the study, these regions (which were abolished in May 2008 with the creation of Alberta Health Services) accounted for over 80% of provincial continuing care beds. These facilities are now referred to as Supportive Living Level 3 or 4 facilities.^4^

Eligible DAL facilities were those that had been in operation for at least 6 months, did not primarily serve residents with mental illness or developmental disabilities, and housed a minimum number of DAL residents at least 65 years of age (≥ 4 for small facilities, ≥ 10 for large facilities). Residents of participating DAL sites were excluded if they were less than 65 years of age, had been recently admitted (< 21 days), were receiving palliative care (expected survival < 6 months), and/or their participation was otherwise deemed inappropriate by staff or family. In addition, a random sample of 1000 long-term care residents from 54 facilities was assessed as part of ACCES. This long-term care cohort, described further in online Appendix A, was included in the current study for comparative purposes. Further details regarding ACCES are published elsewhere.^15^–^17^

Ethics approval was obtained from the University of Calgary Conjoint Health Research Ethics Board, the University of Alberta Health Research Ethics Board, and the University of Lethbridge Human Subject Research Committee.

### Characteristics of residents

At baseline (2006 to 2008), trained research nurses administered the Resident Assessment Instrument for DAL and long-term care residents (using the interRAI for assisted living or long-term care facilities, respectively; see www.interrai.org/instruments.html). These validated instruments provide a comprehensive, standardized assessment of residents' sociodemographic characteristics, physical and cognitive status, health conditions, behavioural problems, and use of medications and services.^18^,^19^

Resident characteristics examined included age, sex, marital status, length of stay in the facility, social engagement, cognitive and functional status, depressive symptoms, health stability, fatigue (defined as inability to complete normal daily activities in past 3 days), aggressive behaviours, number of chronic diseases, number of medications (including hyperpolypharmacy, 20 defined as use of 11 or more medications), falls, hospital admissions in the past year, bladder and/or bowel incontinence, and presence of advance directives. InterRAI-derived scales included the following: Cognitive Performance Scale^21^; Activities of Daily Living Self-Performance Hierarchy Scale^22^; Depression Rating Scale^23^; Changes in Health, End-stage disease and Symptoms and Signs (CHESS) Scale for health instability^24^; and the Aggressive Behavior Scale.^25^ On all scales, a higher score indicates more severe impairment. Comorbidity was measured as the sum of recorded diagnoses on the interRAI instruments. A total of 49 diagnoses were considered in this comorbidity score, capturing chronic health conditions relevant to a resident's current functional and cognitive status, treatment and monitoring needs, and risk of decline in health status. Social engagement was assessed by 2 measures calculated from items on the instruments: strength of social relationships and average time involved in activities when awake and not receiving treatments or assistance with activities of daily living.

### Characteristics of facilities

For each facility, an administrator, manager, or director of care (i.e., someone familiar with the facility who had direct knowledge about the residents) was surveyed approximately midway during follow-up. The facility characteristics examined included the following: location (health region, community size); ownership (for-profit v. not-forprofit; whether part of a chain); year in which spaces opened; availability of other levels of care on site, including long-term care and acute care beds; type and size of facility (number of DAL spaces and total facility spaces); and staffing levels and oversight (availability of licensed practical nurses [LPNs] and/or registered nurses [RNs] on site 24 hours a day, 7 days a week; physician involvement or affiliation with site).

### Outcomes

The primary outcome was time to first admission to an acute care hospital within a year of the baseline assessment. This outcome was determined via linkage with the Alberta Inpatient Discharge Abstract Database. We examined the date of admission, the most responsible diagnosis (based on codes from the International Statistical Classification of Diseases and Related Health Problems, 10th revision, Canadian enhancement [ICD-10-CA]^26^), the length of stay, and alternate level of care (ALC) bed-days (i.e., when a person was occupying a hospital bed but did not require the intensity of resources and services provided in this care setting). We assessed the first discharge event associated with an admission to acute care, rather than total hospital admissions, as the latter may include admissions occurring after a move from the original setting and may reflect characteristics of the new location. This approach captured nearly all residents admitted to acute care (413 [97.4%] of 424 DAL residents and 137 [98.6%] of 139 long-term care residents) during follow- up. Detailed information on other transitions was obtained from facility discharge tracking forms (provided at the time of transfer or death), family caregiver discharge or decedent interviews (performed around the time of transfer or death), and family caregiver interviews at 1-year follow-up (assessing all moves from baseline).

### Analysis

We used descriptive analyses to examine the distribution of DAL resident and facility characteristics overall and by outcome status. We derived the incidence of hospital admission for the DAL and long-term care cohorts, accounting for the occurrence of death as a competing risk using cumulative incidence competing risk (CICR) curves.^27^

We used multivariable Cox proportional hazards models^28^,^29^ to examine the relative importance of resident and facility characteristics as predictors of the time to first acute care admission for the DAL cohort. We accounted for clustering of residents within facilities by calculating robust sandwich standard errors.^30^ Residents were classified into discrete outcome groups according to the date of their first event (i.e., inpatient hospital admission, admission to long-term care or death without prior hospital admission, other transitions without prior hospital admission, no event and remained in DAL throughout the year). Residents were censored on the date of occurrence of long-term care admission (DAL cohort), death, or discharge to some other setting. Those who experienced none of these events and remained in a DAL facility throughout the year were censored on the date of their 1-year follow-up assessment.

Baseline resident and facility characteristics examined as potential predictors of hospital admission were selected on the basis of previous literature.^8^,^12^,^13^,^31^–^36^ Resident-level variables that were significant (*p* < 0.05) in age-adjusted analyses were entered one at a time and were retained if they remained significant predictors (*p* < 0.10) in the full model. We then incorporated health region (fixed effect) and tested the significance of each of the facility-level variables entered separately. Because of relatively high correlations among facility characteristics, we examined separate models testing the effect of each facility variable, adjusting for resident characteristics.^13^

Analyses were conducted using SAS version 9.2 and R version 2.13-1.

## Results

Fifty-nine of the 60 DAL facilities meeting the inclusion criteria agreed to participate. Of the 1510 eligible DAL residents in these facilities, 1089 (72.1%) were enrolled and assessed; of those not enrolled, 339 (22.5% of all eligible residents) refused to participate, and for the remaining 82 (5.4%), the legally designated surrogate could not be contacted. Age and sex were available for 364 (86.5%) of the 421 nonparticipants and showed a similar distribution (mean age 84.4 ± 7.1 years, 74% women) to the age and sex of participants. Of the 1089 participants, 3 had unknown outcomes, and 20 refused consent for administrative data linkage. Therefore, 1066 DAL residents were included in our analyses. Of the random sample of 1000 long-term care residents, 3 could not be linked with administrative data, and 21 did not consent to data linkage; therefore, 976 were included in our analyses.

The DAL residents were typically older widowed women (mean age 84.9 ± 7.3 years, 71.4% widowed, and 76.7% women) (Table 1). The mean number of chronic conditions was 4.7 ± 2.0 (range 0–14), with Alzheimer disease and related dementias (n = 609 [57.1%]), hypertension (n = 604 [56.7%]), arthritis (n = 572 [53.7%]), depression ( n = 3 69 [ 34.6%]), a nd o steoporosis ( n = 338 [31.7%]) being the most common. About one-tenth (n = 109 [10.2%]) were reported to have a "do not hospitalize" advance directive. About two-thirds of DAL residents (n = 663 [62.2%]) resided in a facility with an LPN and/or RN on site 24/7 (Table 2). Additional baseline information appears in Tables 1 and 2.

**Table 1:** Baseline sociodemographic, health, and functional characteristics of residents by outcome event during 1-year follow-up, ACCES-DAL cohort (n = 1066)

Characteristic	Total no.* (% of total)	Outcome; no. (% of row total)†‡			
		Hospital	LTC or death	Still in DAL	*p* value
**Overall**	1066	413 (38.7)	115 (10.8)	534 (50.1)	
**Age, yr**					
Mean ± SD	84.9 ± 7.3	85.2 ± 7.1	86.1 ± 6.5	84.4 ± 7.5	0.045
65–79	268 (25.1)	97 (36.6)	22 (8.3)	146 (55.1)	0.39
80–85	280 (26.3)	110 (39.4)	28 (10.0)	141 (50.5)	
86–89	243 (22.8)	92 (37.9)	32 (13.2)	119 (49.0)	
≥ 90	275 (25.8)	114 (41.5)	33 (12.0)	128 (46.5)	
**Sex**					0.53
Female	818 (76.7)	312 (38.2)	86 (10.5)	418 (51.2)	
Male	248 (23.3)	101 (41.2)	29 (11.8)	116 (47.2)	
**Marital status**					0.77
Widowed	761 (71.4)	293 (38.7)	82 (10.8)	383 (50.5)	
Married or with a partner	156 (14.6)	63 (40.4)	20 (12.8)	73 (46.8)	
Never married, separated, or divorced	149 (14.0)	57 (38.5)	13 (8.8)	78 (52.7)	
**Strength of social relationships§**					0.003
Moderate to high (3–5)	873 (81.9)	332 (38.2)	83 (9.5)	455 (52.3)	
Low to none (0–2)	193 (18.1)	81 (42.2)	32 (16.7)	79 (41.1)	
**Time involved in activities||**					< 0.001
Most (> 2/3 time)	157 (14.7)	54 (34.8)	9 (5.8)	92 (59.4)	
Some (1/3 to 2/3 time)	417 (39.1)	165 (39.6)	32 (7.7)	220 (52.8)	
Little to none (< 1/3 time)	492 (46.2)	194 (39.6)	74 (15.1)	222 (45.3)	
**Cognition (CPS score)**					< 0.001
Intact (0)	223 (20.9)	98 (44.6)	8 (3.6)	114 (51.8)	
Borderline intact (1)	211 (19.8)	82 (38.9)	15 (7.1)	114 (54.0)	
Mild impairment (2)	336 (31.5)	131 (39.1)	31 (9.3)	173 (51.6)	
Moderate to more severe impairment (≥ 3)	296 (27.8)	102 (34.5)	61 (20.6)	133 (44.9)	
**Activities of daily living (ADL score)**					< 0.001
Independent (0)	454 (42.6)	179 (39.6)	13 (2.9)	260 (57.5)	
Supervision required (1)	186 (17.4)	62 (33.5)	26 (14.1)	97 (52.4)	
Limited impairment (2)	126 (11.8)	42 (33.3)	21 (16.7)	63 (50.0)	
Extensive assistance required or dependent (≥ 3)	300 (28.1)	130 (43.5)	55 (18.4)	114 (38.1)	
**Health instability (CHESS score)¶**					< 0.001
Stable (0)	496 (46.5)	165 (33.5)	40 (8.1)	288 (58.4)	
Mild (1)	312 (29.3)	137 (43.9)	31 (9.9)	144 (46.2)	
Mild to moderate (2)	184 (17.3)	74 (40.2)	24 (13.0)	86 (46.7)	
Moderate to high (≥ 3)	74 (6.9)	37 (50.7)	20 (27.4)	16 (21.9)	
**Fatigue (inability to complete ADL in past 3 days)**					< 0.001
None	433 (40.6)	147 (34.2)	37 (8.6)	246 (57.2)	
Minimal	461 (43.2)	181 (39.3)	46 (10.0)	233 (50.7)	
Moderate to severe	172 (16.1)	85 (49.4)	32 (18.6)	55 (32.0)	
**Primary mode of locomotion**					< 0.001
Walks independently	227 (21.3)	71 (31.4)	16 (7.1)	139 (61.5)	
Walks with assistive device	625 (58.6)	249 (40.0)	66 (10.6)	308 (49.4)	
Uses wheelchair or scooter**	214 (20.1)	93 (43.7)	33 (15.5)	87 (40.8)	
**Falls CAP**					0.06
≥ 1 falls/ 90 days	305 (28.6)	129 (42.4)	39 (12.8)	136 (44.7)	
None	761 (71.4)	284 (37.5)	76 (10.0)	398 (52.5)	
**Depressive symptoms (DRS score)**					0.004
Yes (≥ 3)	203 (19.0)	75 (37.1)	35 (17.3)	92 (45.5)	
No (< 3)	863 (81.0)	338 (39.3)	80 (9.3)	442 (51.4)	
**Aggressive behaviour (ABS score)††**					0.06
None (0)	760 (71.3)	305 (40.2)	69 (9.1)	384 (50.7)	
Moderate (1–2)	174 (16.3)	65 (37.8)	23 (13.4)	84 (48.8)	
Severe (3–5)	102 (9.6)	33 (32.4)	16 (15.7)	53 (52.0)	
Very severe (≥ 6)	30 (2.8)	10 (33.3)	7 (23.3)	13 (43.3)	
**No. of chronic conditions**					
Mean ± SD	4.7 ± 2.0	4.8 ± 2.0	4.9 ± 2.1	4.4 ± 1.9	0.003
0–3	323 (30.3)	107 (33.2)	30 (9.3)	185 (57.5)	0.02
4 or 5	398 (37.3)	155 (39.0)	45 (11.3)	197 (49.6)	
≥ 6	345 (32.4)	151 (44.0)	40 (11.7)	152 (44.3)	
**No. of medications**					
Mean ± SD	8.3 ± 3.7	9.1 ± 3.8	8.5 ± 3.6	7.7 ± 3.5	< 0.001
0–6	349 (32.7)	106 (30.5)	36 (10.3)	206 (59.2)	< 0.001
7 or 8	232 (21.8)	88 (37.9)	31 (13.4)	113 (48.7)	
9 or 10	214 (20.1)	87 (41.0)	20 (9.4)	105 (49.5)	
≥ 11	271 (25.4)	132 (48.9)	28 (10.4)	110 (40.7)	
**Advance directive: do not hospitalize**					0.98
Yes	109 (10.2)	42 (39.3)	11 (10.3)	54 (50.5)	
No	957 (89.8)	371 (38.8)	104 (10.9)	480 (50.3)	
**No. of inpatient admissions to hospital in past year**					< 0.001
0	663 (62.2)	228 (34.5)	75 (11.3)	358 (54.2)	
1	254 (23.8)	100 (39.7)	23 (9.1)	129 (51.2)	
≥ 2	149 (14.0)	85 (57.0)	17 (11.4)	47 (31.5)	
**Bladder incontinence**					< 0.001
Continent	436 (40.9)	168 (38.7)	27 (6.2)	239 (55.1)	
Some control, infrequent episodes	156 (14.6)	64 (41.0)	12 (7.7)	80 (51.3)	
Occasional incontinence	114 (10.7)	48 (42.1)	11 (9.6)	55 (48.2)	
Frequent episodes, no control	360 (33.8)	133 (37.2)	65 (18.2)	160 (44.7)	
**Bowel incontinence**					< 0.001
Continent	766 (71.9)	290 (38.0)	66 (8.7)	407 (53.3)	
Some control, infrequent episodes	165 (15.5)	74 (45.1)	16 (9.8)	74 (45.1)	
Occasional incontinence	83 (7.8)	28 (33.7)	20 (24.1)	35 (42.2)	
Frequent episodes, no control	52 (4.9)	21 (40.4)	13 (25.0)	18 (34.6)	

ABS = Aggressive Behavior Scale^25^ (see below), ACCES = Alberta Continuing Care Epidemiological Studies, ADL score = score on Activities of Daily Living Self-Performance Hierarchy Scale,^22^ CAP = Clinical Assessment Protocol, CHESS = Changes in Health, End-stage disease and Symptoms and Signs scale for health stability,^24^ CPS = Cognitive Performance Scale,^21^ DAL = designated assisted living, DRS = Depression Rating Scale,^23^ LTC = long-term care, SD = standard deviation.

* Sample excludes 3 residents with unknown outcome who discontinued the study and 20 who refused consent for linkage of administrative data.

† Except where indicated otherwise.

‡ Four residents (0.4% of the cohort) had other outcomes (censored at date of fi rst discharge from DAL) and were omitted from the comparisons.

§ Social relationships were based on a summary score of items assessing whether the resident was close to someone in the facility, had a strong or supportive relationship with the family, participated in social activities of long-standing interest, and visited or had other interactions with at least one long-standing social relation or family member in the past week.

|| Activity involvement reflected time when the person was awake and not receiving treatments or ADL care.

¶ Two items (insufficient fluid, noticeable decline in food or fluid) that are usually used to calculate CHESS were not included on the interRAI Assisted Living tool.

** Includes 1 resident who was bedbound.

†† The ABS is a summary scale of 4 behaviours (verbal abuse, physical abuse, socially inappropriate or disruptive, resists care), with higher scores indicating a greater number and frequency of behavioural issues.

**Table 2:** Baseline characteristics of care system or facility in relation to residents' outcome events during 1 year follow-up, ACCES-DAL cohort (n = 1066)

Characteristic	Total no.* (% of total)	Outcome; no. (% of row total)†			
		Hospital	LTC or death	Still in DAL	*p* value
**Overall**	1066	413 (38.7)	115 (10.8)	534 (50.1)	
**Region**					0.017 1
1 (urban)	311 (29.2)	111 (35.8)	30 (9.7)	169 (54.5)	
2 (mixed urban/rural)	228 (21.4)	82 (36.1)	31 (13.7)	114 (50.2)	
3 (rural)	153 (14.4)	78 (51.0)	12 (7.8)	63 (41.2)	
4 (urban)	268 (25.1)	96 (36.0)	27 (10.1)	144 (53.9)	
5 (rural)	106 (9.9)	46 (43.8)	15 (14.3)	44 (41.9)	
**Ownership**					0.08
For-profit	420 (39.4)	148 (35.4)	42 (10.0)	228 (54.5)	
Not-for-profit or RHA	646 (60.6)	265 (41.1)	73 (11.3)	306 (47.5)	
**Part of chain**					< 0.001
Not part of chain or RHA-operated	157 (14.7)	76 (48.7)	12 (7.7)	68 (43.6)	
Part of AL chain	334 (31.3)	131 (39.3)	22 (6.6)	180 (54.1)	
Part of AL/LTC chain	575 (53.9)	206 (36.0)	81 (14.1)	286 (49.9)	
**Year DAL spaces opened**					0.65
Before 2002	273 (25.6)	114 (42.1)	24 (8.9)	133 (49.1)	
2002 or 2003	362 (34.0)	135 (37.4)	43 (11.9)	183 (50.7)	
2004 or later	431 (40.4)	164 (38.1)	48 (11.2)	218 (50.7)	
**No. of DAL spaces**					0.002
< 20	109 (10.2)	59 (54.6)	11 (10.2)	38 (35.2)	
20–29	172 (16.1)	77 (44.8)	22 (12.8)	73 (42.4)	
30–39	293 (27.5)	106 (36.4)	29 (10.0)	156 (53.6)	
≥ 40	492 (46.2)	171 (34.8)	53 (10.8)	267 (54.4)	
**Total no. of spaces**					0.15
< 55	148 (13.9)	70 (47.3)	19 (12.8)	59 (39.9)	
55–89	263 (24.7)	104 (39.7)	29 (11.1)	129 (49.2)	
90–147	259 (24.3)	97 (37.6)	29 (11.2)	132 (51.2)	
≥ 148	396 (37.1)	142 (36.0)	38 (9.6)	214 (54.3)	
**Levels of care on site‡**					0.34
DAL only or DAL + equivalent or lower level	859 (80.6)	325 (38.0)	97 (11.3)	434 (50.7)	
DAL + higher level	207 (19.4)	88 (42.7)	18 (8.7)	100 (48.5)	
**LTC beds on site**					0.54
No	865 (81.1)	330 (38.3)	97 (11.3)	435 (50.5)	
Yes (LTC or LTC-dem)	201 (18.9)	83 (41.5)	18 (9.0)	99 (49.5)	
**LPN/RN coverage on site**					0.002
Neither on site	295 (27.7)	138 (46.9)	34 (11.6)	122 (41.5)	
LPN and/or RN < 24/7	108 (10.1)	47 (43.9)	9 (8.4)	51 (47.7)	
LPN and/or RN 24/7	663 (62.2)	228 (34.5)	72 (10.9)	361 (54.6)	
**Physician (GP) affiliated with site**					0.066
No	687 (64.4)	266 (38.8)	87 (12.7)	332 (48.5)	
Yes, office on site	169 (15.9)	63 (37.5)	10 (6.0)	95 (56.5)	
Yes, no office on site	210 (19.7)	84 (40.2)	18 (8.6)	107 (51.2)	
**Community size**					0.007
< 10 000	222 (20.8)	104 (47.1)	18 (8.1)	99 (44.8)	
10 000 – 99 999	292 (27.4)	116 (39.9)	40 (13.7)	135 (46.4)	
≥ 1 million	552 (51.8)	193 (35.1)	57 (10.4)	300 (54.5)	

ACCES = Alberta Continuing Care Epidemiological Studies; AL = assisted living; DAL = designated assisted living; GP = general practitioner; LPN = licensed practical nurse; LTC = long-term care; LTC-dem = long-term care, specialized dementia care bed; RHA = regional health authority; RN = registered nurse; SD = standard deviation; 24/7 = 24 hours a day, 7 days a week.

* Sample excludes 3 residents with unknown outcome who discontinued the study and 20 who refused consent for linkage of administrative data.

† Four residents (0.4% of the cohort) had other outcomes (censored at date of fi rst discharge from DAL) and were omitted from the comparisons.

‡ Equivalent level of care = private AL, residential, respite (not in LTC), community support and transition beds; lower level of care = independent living, lodge, condo; higher level of care = LTC (including respite), acute care.

Relative to long-term care residents (whose characteristics are presented in online Appendix A), DAL residents had stronger social relationships, were more active, and had fewer health issues, cognitive and functional impairments, mood and behavioural challenges, and comorbidities. DAL residents were significantly less likely than long-term care residents to have a reported "do not hospitalize" advance directive (10.2% v. 29.7%) and were significantly more likely than long term care residents to have been admitted to hospital during the year before baseline (37.8% v. 24.5%).

**Figure 1:**
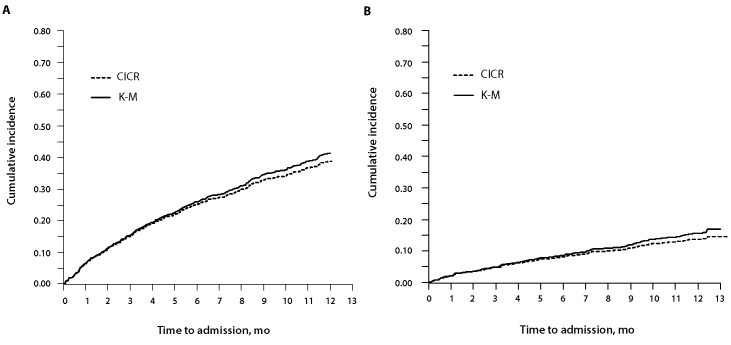
Cumulative incidence of hospital admission during 1-year follow-up. A: Alberta Continuing Care Epidemiological Studies— designated assisted living (ACCES-DAL) cohort (n = 1066). B: Alberta Continuing Care Epidemiological Studies—long-term care (ACCES-LTC) cohort (n = 976). CICR = cumulative incidence competing risk curve, K-M = Kaplan–Meier curve.

During the 1-year follow-up, 413 (38.7%) of the 1066 DAL residents experienced an acute care hospital admission as their first event. The rate was 55.6 per 100 person-years. The cumulative incidence of hospital admission was 25.2% (95% confidence interval [CI] 22.6%–27.8%) at 6 months and 38.9% (95% CI 35.9%– 41.9%) at 12 months (Figure 1A). The median length of stay for these hospital admissions was 12 days (interquartile range [IQR] 5–33 days), and the total number of bed-days was 10 388. Ninety-two (22.3%) of these 413 patients had one or more ALC bed-days (total ALC bed-days = 1907; median ALC length of stay 11.5 days [range 1–96 days]). The proportion admitted to hospital as their first event did not differ between those with and those without a "do not hospitalize" advance directive (42/109 [38.5%] v. 371/957 [38.8%]).

During the 1-year follow-up, 137 (14.0%) of the 976 long-term care residents experienced an acute care hospital admission as their first event. The rate was 17.5 per 100 person-years. The cumulative incidence of hospital admission was 8.0% (95% CI 6.3%–9.7%) at 6 months and 13.7% (95% CI 11.5%–15.8%) at 12 months (Figure 1B), significantly lower than the corresponding values observed for DAL residents (*p* < 0.001 for both). The median length of stay for these admissions was 6 days (IQR 3–11 days), and the total number of bed-days was 1146. One admission (0.7%) had any ALC bed-days (total ALC bed-days = 16). The proportion admitted to hospital as their first event was significantly lower among those who had a "do not hospitalize" advance directive than among those without such an advance directive (26/290 [9.0%] v. 111/686 [16.2%]; *p* = 0.003).

Information on the common reasons for admission to hospital appears in online Appendix B. The corresponding CICR curves stratified by number of previous admissions are presented in online Appendix C.

In adjusted analyses, a significantly greater risk for hospital admission was observed for DAL residents with health instability (as indicated by their CHESS scores), moderate to severe fatigue, hyperpolypharmacy (i.e., use of 11 or more medications), and at least 2 hospital admissions during the preceding year (Table 3). Residents aged 90 years or older and those with poor social relationships had a modestly greater risk. DAL residents from one health region showed a significantly higher risk of hospital admission. Community size was highly correlated with region and was not retained in the models.

**Table 3:** Hazard ratios for hospital admission during 1-year follow-up, ACCES-DAL cohort (n= 1066)

Characteristic	HR (95% CI)	
	Age-adjusted	Fully adjusted*
**Patient-related factors**		
**Age, yr**		
65–79 (reference)	NA	1.00
80–85	NA	1.16 (0.86–1.56)
86–89	NA	1.08 (0.77–1.52)
≥ 90	NA	1.26 (0.97–1.64)†
**Sex**		
Female	0.87 (0.67–1.12)	0.89 (0.68–1.16)
**Strength of social relationships**		
Moderate to high (reference)	1.00	1.00
Low to none	1.21 (0.97–1.50)†	1.22 (0.98–1.52)†
**Average time involved in activities**		
Most (> 2/3 time) (reference)	1.00	
Some (1/3 to 2/3 time)	1.20 (0.92–1.55)	–
Little to none (< 1/3 time)	1.28 (1.00–1.64)	–
**Activities of daily living (ADL score)**		
Independent (0) (reference)	1.00	
Supervision required (1)	0.85 (0.62–1.17)	–
Limited impairment (2)	0.93 (0.61–1.42)	–
Extensive assistance required or dependent (≥ 3)	1.28 (1.06–1.56)	–
**Health instability (CHESS score)**		
Stable (0) (reference)	1.00	1.00
Mild (1)	1.44 (1.16–1.80)	1.26 (1.02–1.57)
Mild to moderate (2)	1.39 (1.03–1.88)	1.16 (0.90–1.49)
Moderate to high (≥ 3)	2.47 (1.64–3.73)	1.65 (1.06–2.56)
**Fatigue (inability to complete daily activities in past 3 days)**		
None (0) (reference)	1.00	1.00
Minimal (1)	1.25 (1.01–1.56)	1.05 (0.86–1.28)
Moderate to severe (≥ 2)	1.97 (1.49–2.61)	1.59 (1.20–2.11)
**Primary mode of locomotion**		
Walks independently (reference)	1.00	
Walks with assistive device	1.40 (1.03-1.88)	–
Uses wheelchair or scooter	1.71 (1.20-2.42)	–
**Falls CAP**		
≥ 1 falls/90 days	1.25 (1.03-1.53)	–
**No. of chronic conditions**		
0–3 (reference)	1.00	1.00
4 or 5	1.28 (1.00–1.64)	1.16 (0.90–1.49)
≥ 6	1.60 (1.25–2.05)	1.23 (0.93–1.62)
**No. of medications**		
0–6 (reference)	1.00	1.00
7 or 8	1.37 (1.04–1.81)	1.29 (0.96–1.72)†
9 or 10	1.50 (1.15–1.97)	1.29 (0.97–1.70)†
≥ 11	2.04 (1.54–2.70)	1.70 (1.31–2.21)
**No. of hospital admissions in past year**		
0 (reference)	1.00	1.00
1	1.23 (1.00–1.49)	1.09 (0.89–1.35)
≥ 2	2.22 (1.78–2.76)	1.86 (1.48–2.35)
**System or facility factors**		
**Region**		
1 (urban) (reference)	1.00	1.00
2 (mixed urban/rural)	1.04 (0.83–1.30)	0.88 (0.70–1.10)
3 (rural)	1.67 (1.28–2.19)	1.55 (1.22–1.98)
4 (urban)	1.00 (0.73–1.38)	0.96 (0.72–1.27)
5 (rural)	1.43 (0.93–2.22)	1.32 (0.84–2.09)
**Community size**		
< 10 000 (reference)	1.00	
10 000 – 99 999	0.80 (0.62–1.04)†	–
≥ 1 million	0.65 (0.50–0.84)	–

ACCES = Alberta Continuing Care Epidemiological Studies; ADL score = score on Activities of Daily Living Self-Performance Hierarchy Scale; CAP = Clinical Assessment Protocol; CHESS = Changes in Health, End-stage disease and Symptoms and Signs scale for health stability; CI = confidence interval; DAL = designated assisted living; HR = hazard ratio; NA = not applicable.

* Derived from Cox proportional hazards regression models (fi rst-event analysis), with adjustment for clustering by facility; sample excludes 3 residents with unknown outcome who discontinued the study and 20 who refused consent for administrative data linkage. A dash indicates that the variable did not remain significant in the fully adjusted model.

† p < 0.10.

In models adjusted for resident characteristics and for health region, a significantly higher likelihood of hospital admission was observed for residents from DAL facilities that were smaller (< 30 DAL spaces or < 55 total spaces), that had no LPN and/or RN on site (or on site less than 24/7), and that were not affiliated with a chain (Table 4).

**Table 4:** Adjusted hazard ratios* for admission to hospital during 1-year follow-up associated with selected facility factors for the ACCES-DAL cohort (n = 1066)

Model†	Adjusted HR (95% CI)
**Model A: no. of DAL spaces**	
< 20	1.79 (1.33–2.42)
20–29	1.29 (1.03–1.61)
30–39	1.00 (0.75–1.34)
≥ 40 (reference)	1.00
**Model B: total no. of spaces**	
< 55	1.49 (1.12–1.97)
55–89	0.97 (0.76–1.24)
90–147	1.06 (0.83–1.36)
≥ 148 (reference)	1.00
**Model C: LPN or RN coverage on site**	
Neither on site	1.42 (1.16–1.73)
LPN and/or RN less than 24/7	1.43 (1.15–1.77)

ACCES = Alberta Continuing Care Epidemiological Studies; AL = assisted living; CI = confidence interval; DAL = designated assisted living; HR = hazard ratio; LPN = licensed practical nurse; LTC = long-term care; RHA = regional health authority; RN = registered nurse; 24/7 = 24 hours a day, 7 days a week.

* Derived from Cox proportional hazards regression models (fi rst-event analysis), with adjustment for clustering by facility; the sample excludes 3 residents with unknown outcome who discontinued the study and 20 who refused consent for linkage of administrative data.

† All models were adjusted for age, sex, strength of social relationships, health instability, fatigue, comorbidity, number of medications, previous hospital admissions, and region.

## Interpretation

To our knowledge, this is the first Canadian study to examine the incidence of hospital admission for assisted living residents relative to those in long-term care. The cumulative incidence of hospital admission over 1 year that we report here (38.9%) was similar to the incidence reported in 2 US studies. Zimmerman et al.^13^ reported a rate of 12.7% per 100-day quarter (46%–51% per year), whereas Hedrick et al.^37^ found that 40.2% of assisted living residents in their study were admitted to hospital at least once over a year.

Admission to a nursing home can lead to a reduction in hospital use.^38^ Whether this is also true for assisted living represents a key policy question in assessing the potential impact on other components of the health care system if assisted living displaces long-term care as a housing option for vulnerable older people. A striking finding of the current study was the lower rate of hospital admission concurrently seen among long-term care residents, notwithstanding their generally worse baseline health (online Appendix A). Relatively low rates of acute care utilization for long-term care residents in Alberta have been previously reported.^39^ It is possible that long-term care residents, their families, and/or their care providers elect not to seek transfer to acute care for changes in health status because of the severity of pre-existing health concerns. Advance planning may be discussed, implemented, and adhered to more systematically within long-term care facilities than in DAL settings. A "do not hospitalize" directive was more commonly found among long-term care residents; furthermore, even when such directives were present for DAL residents, they had no evident impact on the likelihood of hospital admission.

In fully adjusted models, residents with higher levels of health instability (as indicated by the CHESS score), fatigue, medication use, and previous hospital admissions (i.e., at least 2 admissions in the past year) had a significantly higher risk for acute care admission over 1 year. A high CHESS score has previously been shown to be predictive of hospital admission,^40^ whereas continuing care clients who have been high users of hospital care have a higher likelihood of future admission. 41 Fatigue may be functioning as a marker of frailty. These 3 characteristics (CHESS score, prior hospital use, and fatigue) could be used to define a target group for interventions designed to prevent further admissions. Although the dose-dependent relationship between medication use and risk of hospital admission may reflect the relevance of number of medications as a marker of multiple morbidity and/or severity of illness, many hospital admissions of older individuals are drug-related.^42^ Optimizing medication use in assisted living facilities, including prescribing, dispensing, and monitoring of beneficial and adverse effects, has been highlighted as an area requiring improvement.^7^,^43^–^45^

Of the facility-level factors, size of the facility, staffing hours, and staffing mix are also potentially modifiable. Other researchers have shown that a higher proportion of licensed nursing staff hours (whether LPN or RN) or more hours of RN staff time per resident might reduce the risk of hospital admission in residential care and assisted living.^9^,^13^ T he s taffing m odel u sed i n a ssisted living should be commensurate with residents' needs. As Table 1 indicates, the health concerns of these residents are substantial. Our results suggest that greater access to skilled nursing care and other health care professionals may be needed to both monitor for early manifestations of declining health and ensure the capacity to accommodate short-term illnesses on site. Given the correlations among the facility variables examined here, it is difficult to tease out the relative importance or underlying mechanisms associated with these characteristics. Larger size, greater availability of on-site professional staffing, and chain affiliation may contribute to enhanced clinical oversight and services, leading to more timely and effective care.^9^

### Strengths and limitations

The strengths of this study include the large sample, the diverse range of residentand facility-level characteristics examined, and comprehensive, prospective collection of data. However, some limitations warrant consideration. About 28% of eligible DAL residents were not enrolled. Although the demographic characteristics of these nonparticipants were similar to those of participants, nonparticipation by some residents may limit the generalizability of our findings. Our study was restricted to residents of publicly subsidized assisted living spaces in Alberta, as these settings are subject to provincial care standards and admission is through a single point of entry. Although some caution is warranted in generalizing our results to private-pay institutions or to assisted living facilities in other provinces, all assisted living settings share common elements that differentiate them from long-term care. Alberta has been a trendsetter in exploring the role of assisted living within Canada, and other provinces currently considering an expansion of assisted living settings for the care of vulnerable older people can learn from its experiences. Finally, data collection took place between 2006 and 2009, and changes have taken place within the Alberta assisted living sector since then. The possible effect of these changes on rates of hospital admission is unknown.

### Conclusion

Nearly 40% of DAL residents in Alberta were admitted to hospital over 1 year, a rate substantially higher than that for long-term care residents. The risk of hospital admission was associated with a number of characteristics that could be targeted for improvement (e.g., health instability, frequent prior admissions, fatigue) and/or used for developing interventions (e.g., optimizing medication use, staffing). A shift toward assisted living from long-term care for the supportive care of vulnerable older people, as proposed in both Alberta^46^ and Ontario,^47^ could lead to an increase in the demand for hospital beds. Our study does not indicate the "correct" rate for hospital admissions in this vulnerable population, but we believe that a proportion of the admissions for DAL residents were potentially preventable. Avoiding such admissions would protect DAL residents from the negative consequences associated with a hospital stay and would mitigate the attendant costs and inefficiencies arising from inappropriate use of hospital beds.
